# Present and future of the undergraduate ophthalmology curriculum: a survey of UK medical schools

**DOI:** 10.5116/ijme.59ac.f69b

**Published:** 2017-11-02

**Authors:** Sophie Hill, Reg Dennick, Winfried Amoaku

**Affiliations:** 1 Ophthalmology Department, Nottingham University Hospitals NHS Trust, Nottingham, UK; 2 Medical Education Unit, University of Nottingham, Nottingham, UK; 3 Academic Ophthalmology, Division of Clinical Neurosciences, University of Nottingham, UK

**Keywords:** Ophthalmology, undergraduate, curriculum, UK

## Abstract

**Objectives:**

To investigate the current undergraduate ophthalmology curricula provided by the UK medical schools, evaluate how they compare with the guidelines of the Royal College of Ophthalmologists (RCOphth) and International Council for Ophthalmology (ICO), and determine the views of the UK ophthalmology teaching leads on the future direction of the curriculum.

**Methods:**

A cross-sectional questionnaire was sent to teaching leads in 31 medical schools across the UK. The questionnaire evaluated eight themes of the curriculum: content and learning outcomes, communication of learning outcomes, organisation of the curriculum, assessment, educational resources, teaching methods used, and the educational environment. The ophthalmology teaching leads were also asked their opinion on the current and future management of the curriculum. These were compared with RCOphth and ICO guidelines and descriptive statistical analysis performed.

**Results:**

A response rate of 93% (n=29/31) was achieved. The knowledge and clinical skills taught by the UK medical schools match the  RCOphth guidelines, but fail to meet the ICO recommendations. A diverse range of assessment methods are used by UK medical schools during ophthalmology rotations. Variation was also observed in the organisation and methods of ophthalmology teaching.  However, a significant consensus about the future direction of the curriculum was reported by teaching leads.

**Conclusions:**

Comprehensive RCOphth guidance, and resource sharing between medical schools could help to ensure ophthalmology’s continuing presence in the medical curriculum and improve the effectiveness of undergraduate ophthalmology teaching, while reducing the workload of local teaching departments and medical schools.

## Introduction

Debate about ophthalmology’s place within the undergraduate medical curriculum has been ongoing for nearly a hundred years.^1^ The explosive growth of scientific discovery has created relentless pressure to include novel subjects in the undergraduate medical curriculum. These changes have led to the erosion of more traditional subjects, including ophthalmology.^2^^-^^4^

The earliest reference to an undergraduate ophthalmology curriculum was in 1919, when the Council of British Ophthalmologists issued a report on the ‘Teaching and Examination of Medical Students in Ophthalmology’.^1^ The undergraduate ophthalmology curriculum described in this report concentrated only on the duration of the ophthalmology teaching and the assessment of knowledge. No reference is made to the content of the curriculum or teaching methods or environment. Despite the brevity of this report, the influence that the British Council of Ophthalmology held resulted in the General Medical Council recommending that at least ten weeks of ophthalmology training was undertaken during the undergraduate course.^1^ These recommendations are dramatically different to those of the current General Medical Council. With the publication of ‘Tomorrows Doctors’ ^5^ the UK undergraduate medical education system underwent major changes. More recent guidelines outlining the concept of a core undergraduate medical curriculum make no reference at all to the speciality of Ophthalmology.^6^ As a result ophthalmology undergraduate rotations have become more vulnerable in the UK.

Ophthalmology specialists have been comparatively peripheral to the education and training of doctors, such that ophthalmology has become isolated in the undergraduate medical curriculum.^7^ A larger emphasis on the learning of clinical skills in general practice rotations, rather than specialty attachments has reduced dedicated ophthalmology teaching.^4^ Conflict about what the end product of an ophthalmology rotation should achieve: a general practitioner, researcher or specialist has also created further confusion about the content of the curriculum.^4^ Despite this decline in undergraduate ophthalmology teaching, the ability to perform direct ophthalmoscopy examination is still an expected competency during UK Foundation Year One training.^8^

Between 7-19% of Accident and Emergency presentations have an ophthalmic component.^9^^,^^10^ In addition, it is estimated that 2-5% of all consultations in primary care are for ophthalmic conditions.^11^^,^^12^ These patients may present with sight threatening and potentially life-threatening illnesses. Therefore, it is critically important that junior doctors and general practitioners have the knowledge and skills required to treat and refer ophthalmic patients. This is especially relevant in the UK population where there is a rising prevalence of Type 2 diabetes with a resultant increase in diabetic retinopathy.

Several studies have highlighted the lack of confidence reported by clinicians when dealing with eye conditions.^13^^, ^^14^ It was suggested that if undergraduate ophthalmological teaching could be made more effective, the standard of primary ocular care would improve and have beneficial consequences for the population and for the ophthalmologist.^2^

In 2006, the International Task Force on Ophthalmic Education of Medical Students on behalf of The International Council of Ophthalmology (ICO) published the ‘Principles and Guidelines of a Curriculum for Ophthalmic Education of Medical Students’^15^ to try and address some of these challenges.

The ICO suggests that an evidence based ophthalmic curriculum should be included in the core curriculum of all medical schools. The undergraduate curriculum should also contain the basic skills and knowledge needed for satisfactory primary eye care, and ophthalmic instruction should enable students to recognise the ocular manifestations of systemic disease and when to refer to ophthalmology.^15^ During the report the elements of the curriculum are divided into hours in the curriculum, specific teaching methods, resources, and content. Forty to sixty hours (or 5 to 8 days) duration is recommended. Specific teaching methods such as lectures, clinical placements, case studies and integration with other specialities (e.g. neuroscience, neurology, endocrinology, and geriatric medicine) are also advocated. The suggested content of the curriculum consists of an extensive list of learning objectives. The knowledge topics are broadly divided into twelve categories of “fundamentals and principles of ophthalmology, cornea and external diseases, lens and cataract, neuro-ophthalmology, vitreoretinal disease, glaucoma, paediatric ophthalmology and strabismus, diseases of the eyelid lacrimal system and orbit, ocular manifestation of systemic disease, intraocular tumours, refraction and contact lens, and refractive surgery”.^16^ Eleven clinical skills are also defined these are “visual acuity, external inspection, pupillary reaction testing, pupillary dilation, ocular motility, direct ophthalmoscopy, intra-ocular pressure measurement, anterior chamber depth assessment, confrontation field testing, upper lid eversion, fluorescein staining of the cornea”.^16^

This comprehensive guidance has formed the template for the minimum standards of ophthalmic undergraduate internationally. Thus, many studies have used the ICO guidance as a benchmark from which they evaluate and compare their own curriculum.

In the UK, the Royal College of Ophthalmologists (RCOphth) provides a recommended undergraduate curriculum. A brief outline of the recommended ophthalmic diagnostic skills and knowledge that a medical student should attain is described. Knowledge domains include:

“Competence and understanding in basic ophthalmic history taking and examination; the ability to differentiate common causes of red eye and understand their management; differentiate common causes of sudden and gradual loss of vision and understand their management; appreciate common ophthalmological manifestations of general medical conditions; understand how visual impairment impacts on activities of daily living; understand the importance of ocular public health issues, both in the UK and internationally”.^17^  

Their recommended clinical skills are, visual acuity measurement, pupil assessment, confrontation visual fields, cover test and assessment of extraocular movement, and direct ophthalmoscopy.^17^ In 2016 the RCOphth updated their undergraduate curriculum in response to our preliminary report.^18^ 

Several studies have examined what the undergraduate ophthalmology curriculum should contain.^19^^-^^26^ In the last decade, research focus has been on the evaluation of the existing undergraduate ophthalmology curriculum as a method of aiding national curriculum development.^27^^-^^29^ The multiple factors that have to be considered in such curriculum evaluations include the educational environment, educational strategies, learning opportunities, content, learning outcomes, and assessment.^30^

By using the ICO guidance as a framework, we have the opportunity to compare the results of our current study to those of previous studies that had used the ICO guidance to evaluate curriculum content both within the UK,^28^^,^^29^and abroad.^27^^,^^31^

The aim of this research was to investigate the current undergraduate ophthalmology curricula provided by the UK medical schools, compare them with the RCOphth and ICO guidelines, and determine the views of the undergraduate teaching leads in ophthalmology on the future direction of the curriculum. Eight themes on the undergraduate ophthalmology curriculum were explored, based on the framework of Harden’s ten questions of curriculum development. ^32^

## Methods

### Design and study participants

This was a cross-sectional web based questionnaire. Participants included were the teaching leads for ophthalmology from each UK medical school. Two UK medical schools offer only pre-clinical courses and therefore do not include ophthalmology in their course. These medical schools were excluded from the study.  A total of 31 medical schools were, therefore, invited to take part in the study. The ophthalmology teaching leads representing 29 medical schools responded to the questionnaire.

### Data collection method and procedure

A web-based questionnaire was created using the Bristol Online Survey tool, after ethical approval was granted by the University of Nottingham Faculty of Medicine and Health Sciences, Research Ethics Committee. This questionnaire was modified from a survey designed by Baylis et al^28^ after permission and copyright was obtained from Informa Healthcare publishing group.

The questionnaire explored eight themes of curriculum development. These included, content and learning outcomes of the curriculum, how those learning outcomes are communicated to students, organisation of the curriculum and the teaching methods used, assessment, educational resources, and the educational environment. The opinions of the undergraduate ophthalmology teaching leads on the current and future management of the curriculum were also explored with Likert scale questions and free text responses. The categories of the knowledge and skills were taken from the pre-2016 Royal College of Ophthamologists guideline^17^ and the International Council of Ophthamology’s guideline on the undergraduate curriculum.^15^

After an initial pilot study the web based survey was distributed to all undergraduate ophthalmology teaching leads via an email link to a Bristol Online Survey webpage. Participants were given two months to respond to the questionnaire. Any respondents that had not replied by the complete by date were followed up and contacted via email to encourage a greater response rate. Responses were collated on the Bristol Online Survey website.  Missing data was identified and a few individuals were contacted directly via email to clarify these answers to increase the data reliability.

### Statistical analysis

The quantitative data collated was analysed using SPSS version 21. Frequency tables and boxplots were produced using SPSS. The data from the free text responses were analysed thematically to determine response categories.

## Results

### Content of the curriculum and communication of learning outcomes

Twenty-seven (96%) of the UK medical schools surveyed had an agreed undergraduate ophthalmology curriculum with twenty-two (76%) of the medical schools reporting defined learning outcomes.  An ophthalmologist is involved in the design of the curricula in 93% (n=26) of these medical schools.

The clinical ophthalmic skills taught by the UK medical schools also adhered to the RCOphth guidelines^33^ but failed to encompass all the skills described in the ICO guidelines.^15^ Only seven of the eleven ICO recommended clinical skills are taught by 75% (n=21) of the UK medical schools, namely visual acuity, pupil reactions, red reflex assessment, direct ophthalmoscopy, confrontation field testing, ocular motility, and the cover test.

**Figure 1:**
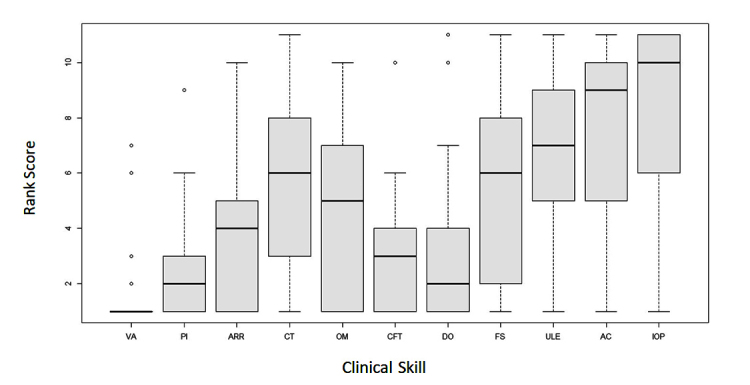
Boxplot graph showing the ophthalmology undergraduate leads ranking score for the importance of each knowledge topic in the curriculum. 1 representing the most important topic to 14, the least important topic (n=29); Key: OA- Ocular anatomy/physiology; RE- Acute red eye; OE-Ocular emergencies; SLV-Sudden loss of vision; LC-Lens and cataract, G- Glaucoma; MR-Medical retina/ macular degeneration; C- Cornea and external disease; VR- Vitreoretinal diseases; EM- Eye manifestations of systemic disease; N- Neuro-ophthalmology; P- Paediatric ophthalmology; IOT-Intraocular tumours; RCE- Refractive and corrective errors.

**Figure 2:**
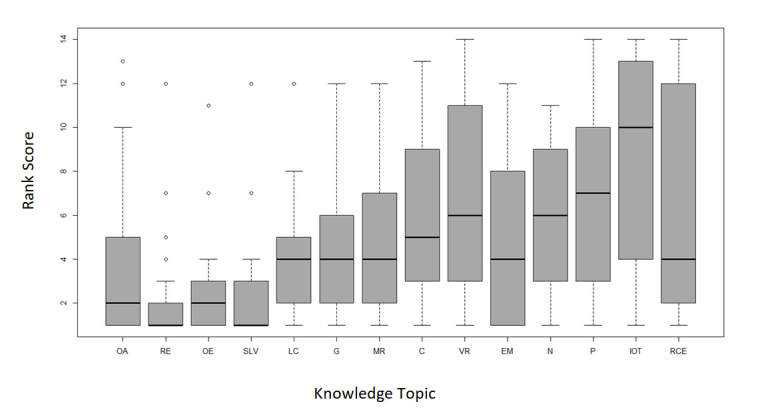
Boxplot graph showing the ophthalmology undergraduate leads ranking score for the importance of each clinical skill in the curriculum. 1 representing the most important clinical skill to 14, the least important clinical skill (n=29); Key: VA-Visual acuity; PI- Pupil reactions; ARR-Assessing red reflex; CT-Cover test; OM- Ocular motility; CFT-Confrontation field testing; DO-Direct ophthalmoscopy; FS- Fluorescein staining of cornea; ULE- Upper lid eversion; AC-Anterior chamber depth assessment; IOP- Intraocular pressure assessment.

The undergraduate teaching leads considered knowledge about the different causes of red eye, sudden loss of vision, and assessment of visual acuity to be the most essential knowledge and skills to be included in the undergraduate course. Figures 1 and 2 show the ranking of the knowledge and skills by importance from 1 to 14.

Knowledge about intraocular tumours and the assessment of intraocular pressure were considered to be the least essential knowledge and skills in the curriculum. These were also found to be the least widely taught knowledge topic and skill by the UK medical schools (Figure 1 and Figure 2).

The most widespread method of communicating learning outcomes to students is via E-learning platforms such as Moodle, followed by study guides.

### Organisation of the curriculum

The organisation of the undergraduate ophthalmology curricula varied across the UK medical schools. Currently, nineteen medical schools provide a stand-alone attachment in ophthalmology of between one and two weeks’ duration. Half of the medical schools provided a one-week attachment, and a further five provided two weeks of ophthalmology teaching. Thirty-one percent (n=9) of the medical schools integrate ophthalmology with other subjects. These subjects included Neurology, Ear, Nose and Throat, Surgery and General Practice.  An additional or optional student selected component is provided by twenty-five of the medical schools, these consist of a wide variety of formats. Student selected attachments/electives are widely available at the UK medical schools for students who wish to extend their experience in ophthalmology. These were commonly 4 weeks in duration with opportunities in research and audit, available in the third, fourth and fifth year of the medical course.

### Teaching methods and educational environment

UK medical schools use various teaching methods in their undergraduate ophthalmology curriculum. The most commonly used teaching method was lectures (96%, n=28), closely followed by self-directed learning (83%, n=24) and small group teaching (82%, n= 24) (Table 1).  An increasing number of medical schools (70%, n=21) used E-learning methods.

**Table 1:** Frequency table showing the teaching methods used by the UK medical schools during the ophthalmology undergraduate curriculum (n=29)

Teaching Methods	N (%)
Lectures	28 (96.5)
Small group	24 (82.7)
1:1/1:2^*^	15 (51.7)
Problem based learning	14 (48.2)
Self-directed learning	24 (82.7)
E-learning	21 (72.4)
Simulation	10 (34.4)

*1:1 represents one tutor for one student; 1:2 represents one tutor for two students

The clinical environments used to provide ophthalmology clinical experience were predominantly outpatient clinics, eye casualty and ophthalmic theaters. When participants were asked to rate whether teaching was given a priority in their departments (curriculum environment), 68% (n=20) agreed and strongly agreed that it was.

**Table 2:** Frequency table showing the assessment methods used by the UK medical schools during the undergraduate ophthalmology curriculum (n=29)

Assessment methods	N (%)
Ophthalmology written exam	11 (44)
Ophthalmology practical skills exam (OSCE)	18 (72)
Inclusion in written finals exam	16 (64)
Inclusion in clinical skills finals exam	17 (68)
Case presentations during ophthalmology rotation	10 (40)
Completion of ophthalmology workbook/logbook	13 (52)
Other	2 (8)

### Assessment of curriculum

Ophthalmology is formally assessed by twenty-five medical schools during their curricula. Of these, 62% (n=18) had an ophthalmology practical skills assessment. However, only 55.1% (n=16) of medical schools included ophthalmology in their final written and 58.6% (n=17) in their final clinical skills examinations (Table 2).

### Curriculum resources

A variety of resources are available to students during their ophthalmology placements. Eighty-nine percent (n=25) of medical schools provided clinical skills facilities for students to practice on simulated clinical models. Online resources and course handouts are also widely reported. Online learning modules were also used by 78% (n=22) of UK medical schools.

### Respondents’ opinions about the current and future management of the undergraduate ophthalmology curriculum

The final part of the survey focused on the opinions of the teaching leads on the current and future management of the undergraduate ophthalmology curriculum. Twenty-one respondents knew of the RCOphth undergraduate curriculum and its content. All respondents agreed that having an RCOphth curriculum to follow was vital, important, or useful. Ninety-six percent (n=27) of respondents also agreed that the RCOphth should have resources for teaching. More financial support was considered vital by 62% (n=18) while 78% (n=22) felt that more time for teaching was important or vital. No consensus was reached on the ideal duration of the undergraduate ophthalmology rotation; six respondents suggested 2 weeks, a further six suggested 4 weeks and three suggested 3 weeks. The remaining respondents suggested a range from four days to eight weeks duration. Despite these differences in opinion, all the respondents agreed that their teaching commitment should be recognised in their job plans as programmed activity (PA) sessions.  Twenty-eight (28) (96%) of respondents were also prepared to share lectures or teaching resources with other UK medical schools.

## Discussion

There have been several studies aimed at establishing what content the undergraduate ophthalmology curriculum should include.^2^^,^^20^^,^^21^^,^^23^^-^^26^ Several national and international curriculum guidance documents have also been drawn up over the years.^15^^,^^34^

In this study the ICO’s undergraduate curriculum guidelines15 were adapted to evaluate and compare what content was currently being taught at the UK medical schools. The ICO guidance was chosen as it provided an opportunity to compare the results of the current study to studies that had used the ICO guidance previously as benchmark to evaluate curriculum content both within the UK^28^^, ^^29^ and abroad.^27^^,^^31^

This study found that a higher percentage of UK medical schools taught each knowledge topic than had previously been reported.^27^^-^^29^^,^^31^ This may be because of a different sample population included in the current survey. However, it may also suggest a greater adherence to the ICO guidelines on the knowledge content of the curriculum is slowly emerging across the UK.

In contrast, the ICO recommended clinical skills are not widely taught. Previous UK studies have reported similar findings with less than 30% of medical schools teaching upper lid eversion, anterior chamber depth assessment, and intraocular pressure assessment.^28^^,^^35^  Reasons for this disparity in the taught clinical skills is unclear. Perhaps, the short curriculum duration in some schools does not allow sufficient time to ensure these skills can be taught. The individual educators’ attitudes towards the necessity of certain skills in the curriculum could also have an influence on their inclusion in the curriculum.

Opinions on the importance of knowledge topics and clinical skills to be included in the undergraduate curriculum vary. This is reflected in the large box plots in Figures 1 and 2. Previous studies agree that red eye, glaucoma, squint, visual failure, and the eye in systemic disease should be included.^2^^,^^21^^,^^24^^-^^26^ However, in the study by Spivey in 1971, the eye in systemic disease was not included.^20^ Clinical skills have also been ranked in previous studies.^26^ Visual acuity ranked the highest followed by pupillary reflexes, ophthalmoscopy and visual field testing, whilst intraocular pressure assessment was considered to be the least important skill.^21^ These results, very closely reflect the opinions of the respondents from this current study.

In their guidelines, the ICO does not consider being able to test the IOP of a patients’ eye as an essential skill.^15^ However, it is suggested that the student should know how IOP measurement is done and be given the opportunity to develop this skill, for example during an elective or student selected component in ophthalmology. It seems that uniformity of opinion on the content of the undergraduate ophthalmology curriculum is unlikely to be achieved. The ICO guideline^15^ provides a compromise on this issue and as such have been widely accepted.^27^^-^^29^^,^^31^^,^^35^^-^^37^

The communication of ophthalmic learning objectives has been rarely mentioned in the literature. Previous suggestions included handouts with checklists of conditions.^24^ This present study has identified the adoption of E-learning platform, such as Moodle, as viable method for communicating learning outcomes to students.

The number of medical schools providing standalone attachments in ophthalmology has significantly increased from the 29% previously reported.^28^ These previous studies suggested much greater levels of integration of ophthalmology teaching with other subjects.^27^^,^^28^ These differences may reflect curriculum organisational change over time in UK undergraduate teaching, and if properly used may increase emphasis on ophthalmology.

There is a high variability in the duration of ophthalmology placements across the UK. If we assume a day provides seven hours of ophthalmology teaching, then most students are receiving approximately 49 hours of ophthalmology teaching exposure. This is not dissimilar to the recommended duration of 40-60 hours (5-8 days) by the ICO.^15^ Previous studies report similar average durations of 7,^31^ 7.6,^28^ 8,^29^ and 8.9 days.^27^ The widespread provision of student-selected components may enable enthusiastic students to pursue their interest in ophthalmology and gain further experience and time in the specialty. However, these attachments are only available to a select number of students, and their uptake will greatly depend upon the quality of their initial ophthalmology attachment. This is a view shared by other authors.^4^^,^^10^^,^^38^

The various teaching methods utilised including a large proportion of e-learning may be a result of the limited time for the curriculum, combined with a greater recognition that computer assisted learning is effective and time efficient.^36^

The positive undergraduate ophthalmology educational environment reported in this study parallels the results of Baylis et al, who reported that most eye departments supported teaching by reducing clinic numbers in order to facilitate education.^28^ These results imply that UK hospital departments involved in undergraduate ophthalmology education regard teaching as valuable and worthwhile.

There remains, however, large variation throughout the UK in the assessment of the undergraduate ophthalmology curriculum. Welch and Eckstein have previously reported a similar level of clinical skills assessment.^29^ Baylis et al (2011),^28^ found comparable levels of assessment of ophthalmology knowledge as in other examinations, but fewer schools reporting ophthalmology clinical skills assessments. Only about a third of medical schools require students to pass their ophthalmology assessments in order to complete the year.^29^ A similar picture is seen in Canada where only 43% of schools count an ophthalmology evaluation towards a students’ assessment record.^39^ How the curriculum can be most effectively assessed remains largely unstudied.

The almost universal adoption of simulation by medical schools to practice ophthalmic clinical skills has occurred recently. Such simulation has been previously adopted in ophthalmology teaching with reports of ophthalmoscopy practice mannequins in Canada in 1998.^39^ This should be encouraged as it allows students to practice their skills prior to examining patients. However, such simulation should not be considered as adequate replacement to patient examination.

This study has shown significant agreement between the respondents about the future direction of the undergraduate ophthalmology curriculum. Closer collaboration between medical schools to improve educational standards and reduce local workload has been proposed previously in Australasia,^27^ America,^40^ and Canada.^31^^,^^39^

Our results highlight the direction that the undergraduate curriculum could take in the future to survive in the crowded undergraduate medical course. Areas for possible resource sharing could include clinical case vignettes, e-learning modules and a database of exam questions. The findings from this study were shared with the RCOphth  Education Committee in 2015. We welcome the launch of a new curriculum by the RCOphth in 2016 “Eyes & Vision Curriculum” for Undergraduate and Foundation Doctors.^18^

### Limitations

The data collected from this questionnaire is limited as only a snap shot of the undergraduate ophthalmology curriculum can be produced from a cross-sectional survey. The sample size of this study was small. However, this sample reflects the individuals responsible for the undergraduate ophthalmology curriculum across the UK. These individuals are therefore best placed to report on the curriculum. With a response rate of 93% (n=29/31), the data collected is likely to be representative.

Attempts were made to reduce measurement error. However, misinterpretation of the survey questions by some of the participants may have resulted in variation in the ranking of the importance of knowledge and skills essential to the curriculum. Asking respondents to organise priorities where there are more than five options to rank, has been previously reported as overwhelming.^41^

The final part of the survey asked the respondents their opinions on the curriculum using a rating scale. This type of question allows a relative degree of preference to be documented.^41^  These types of questions can however be open to interpretation as one participant’s ‘agree’ may be another respondent’s ‘strongly agree’.

## Conclusions

As the most widely accepted standard on the content of the undergraduate ophthalmology curriculum the ICO guideline should be used as a framework to develop a more comprehensive RCOphth UK undergraduate ophthalmology curriculum.

Improved adherence to the ICO guideline would ensure greater standardisation of ophthalmology teaching across the UK. A formal edict from the RCOphth about the organisation, duration and content of the undergraduate ophthalmology curriculum, would assist the undergraduate teaching leads in ensuring ophthalmology’s continuing presence in the undergraduate medical curriculum. Resource sharing between the medical schools facilitated by the RCOphth, could also improve the effectiveness of undergraduate ophthalmology teaching while reducing the workload of local teaching departments and medical schools.

### Acknowledgements

The authors would like to thank Oliver Baylis for sharing his questionnaire proforma; also, Eric Barnes, Daniel Byles, Phillip Murray, and Narciss Okhravi for their advice on the pilot version of the questionnaire. The authors would also like to thank the undergraduate ophthalmology teaching leads for their support in completing the study questionnaire.

### Conflict of Interest

The authors declare that they have no conflict of interest.
